# *Streptomyces luridus* So3.2 from Antarctic soil as a novel producer of compounds with bioemulsification potential

**DOI:** 10.1371/journal.pone.0196054

**Published:** 2018-04-23

**Authors:** Claudio Lamilla, Douglas Braga, Rui Castro, Carolina Guimarães, Livia V. A. de Castilho, Denise M. G. Freire, Leticia Barrientos

**Affiliations:** 1 Laboratory of Applied Molecular Biology, Center of Excellence in Translational Medicine, Temuco, Chile; 2 Scientific and Technological Bioresource Nucleus (BIOREN), Universidad de La Frontera, Temuco, Chile; 3 Laboratório de Biotecnologia Microbiana, Departamento de Bioquímica, Instituto de Química, Universidade Federal do Rio de Janeiro, Av. Athos da Silveira Ramos, Centro de Tecnologia, Cidade Universitária, Rio de Janeiro RJ, Brasil; Universita degli Studi di Milano-Bicocca, ITALY

## Abstract

The present study aimed to identify novel microbial producers of bioemulsificant compounds from Antarctic soils. Fifty-nine microbial strains were isolated from five different locations at South Shetland Islands, Antarctica, and screened for biosurfactant production by β-hemolytic activity. Strain So 3.2 was determined as bioemulsifier-producer and identified by phenotypic and molecular characterization as *Streptomyces luridus*. Emulsification activity, oil displacement method and drop-collapsing test were performed to evaluate the biosurfactant activity with different oils and hydrocarbons using two different culture media (Luria Bertani and Bushnell Haas in the presence of different carbon sources: glucose, glycerol, olive oil and n-Hexadecane). Cell free supernatant of Bushnell Haas culture supplemented with n-Hexadecane showed the best results for all tests. Emulsification of hydrocarbons exceeded 60%, reaching up to 90% on oil with high API grade, while displacement tests ranged from 8 cm to 4 cm in diameter according the culture media and tested oils. Our results revealed that *Streptomyces luridus* So3.2 is able to produce bioemulsifiers capable of emulsifying hydrocarbons and oils, which could be used in different biotechnological applications, particularly for bioremediation of environments contaminated by oil leaks.

## Introduction

Due to its specific environmental conditions, Antarctica is one of the less populated and extreme habitats on Earth [1]. However, life on this continent has not been affected, especially with bacterial microbiota, which is present in all Antarctic environments [2]. In the past few years, the study of Antarctic ecosystems and their microorganisms has received increasing attention [3]. Recently, the biotechnological potential of Antarctic microorganisms has been recognized, particularly regarding secondary metabolites produced by bacteria [4–9]. Among them, biosurfactant molecules of great interest produced by different Antarctic bacteria have been described [10]. Microbial bioprospecting conducted in Antarctica has been focused on different water, soil, and especially marine sediment samples from research stations located on islands such as King George Island (Arctowski Base), Livingston Island (Byers Peninsula), and Budd Shore (Casey Station). Antarctic microorganisms reported as biosurfactant producers include bacterial genera *Pseudomonas* [5], *Bacillus* [10], *Pantoea* [11], *Rhodococcus* [12] and the yeast species *Candida antarctica* [13].

During the last decade, Actinobacteria phylum have been of special interest for their abundance in the soil and their ability to generate bioactive metabolites, useful in various biotechnological, industrial and pharmaceutical processes [14–16]. Within this group, a particular genus that stands out for its variety of secondary metabolite applications is *Streptomyces*, composed of Gram-positive filamentous bacteria [17]. Since *Streptomyces* sp. produces different biomolecules, the production of active surface biomolecules has also been reported, including biosurfactants. As an example, extracellular production of streptofactin, a hydrophobic peptide compound from *Streptomyces tendae*, was reported [18], as well as glycolipids in *Streptomyces matensis* and *Streptomyces coelicoflavus*. Moreover, *Streptomyces* strains isolated from Antarctic soils have been described for their production of biosurfactants, as described by Gesheva and Negoita, where glycolipids were produced by strains isolated from Haswell Island, Antarctica [18].

The terms biosurfactant and bioemulsifier have often been used interchangeably to describe surface-active biomolecules. However, as described previously by Uzoigwe et al. (2015), there are marked differences between them, especially based on their physical-chemical properties and physiological roles. Even though bioemulsifiers and biosurfactants are both amphiphilic in nature and are produced by a wide range of microorganisms, each compound exhibits specific roles in nature [19]. Biosurfactants are classified according to their biochemical nature, such as glycolipids, lipopeptides/lipoproteins or fatty acid/polymer phospholipids [20,21]. They are amphipathic molecules with both hydrophilic and hydrophobic moieties that partition preferentially at the interface between fluid phases with different degrees of polarity, such as oil/water or air/water interfaces. This physical-chemical characteristic allows them to reduce surface and interfacial tension and form microemulsion, where hydrocarbons can solubilize in water, or water can solubilize in hydrocarbons [22]. On the other hand, bioemulsifiers, generally have a higher molecular weight comparing with biosurfactants as they are complex mixtures of heteropolysaccharides, lipopolysaccharides, lipoproteins and proteins [23]. They are also known as high molecular weight biopolymers or exopolysaccharides. Similar to biosurfactants, these molecules can efficiently emulsify two immiscible liquids such as hydrocarbons or other hydrophobic substrates even at low concentrations, but they are less effective at surface tension reduction. Therefore, bioemulsifiers are characterized by their emulsifying activity rather than surfactant activity, which is possible due to the hydrophilic and hydrophobic groups that confer an amphipathic nature to these molecules [24]. Bioemulsifications exhibit a great variety of associated properties due to the innumerable combinations of hydrophilic and hydrophobic residues that molecules can present [25]. For example, surface-active biomolecules can stabilize emulsions by increasing their kinetic stability. This stabilizing property has increased bioemulsifiers value in cosmetics, food, pharmaceutical, and petroleum industries [26–28]. As shown in Table 1, there are multiple current applications of bioemulsifiers isolated from bacterial strains belonging to the *Streptomyces* genus. In addition, these bioactive compounds have shown several interesting properties, such as low toxicity and high biodegradability, digestibility, foaming capacity, selectivity and specificity at extreme temperatures, pH, and salinity [29–31].

**Table 1:** Characteristic of bioemulsifiers from *Streptomyces* genus.

N	ID strains	Carbon sources	Properties	Applications	Chemical structures identified	Site of isolation	Reference
1	*Streptomyces* sp	Sugarcane vinasse	Bioemulsifier	Bioremediation	ND	Contaminated soil in Patagonia, Argentina	Colin et al. [32]
2	*Streptomyces* sp	Glucose	Bioemulsifier	Remediation of organic and inorganic pollutants	Glycoprotein	Collection	Colin et al. [33]
3	*Streptomyces rimosus*	Rapeseed oil	Emulsification	Industrial and non-industrial	Oxytetracycline	Collection	Elsayed et al. [34]
4	*Streptomyces* sp. *S22*	Sunflower oil	Bioemulsifier	Formulation of pesticides,food, pharmaceutical and medicine	Peptidoglycolipid	Garden soil	Maniyar et al. [35]
5	*Streptomyces* sp. *S1*	Toluene	Bioemulsifier	Formulation of pesticides, food and medicine	Polysaccharide	Goa coastalsediment	Kokare et al. [36]
6	*Streptomyces* spp.	Sunflower oil	Bioemulsifier and biosurfactant	Bioremediation of hydrocarbon pollutants	ND	Soli Vellore district	Deepa et al. [37]

ND: not identified

The objective of the present study was to evaluate the capacity of microbial Antarctic isolates for the production of bioemulsifiers compounds, and particularly to characterize the bacterial strain *Streptomyces luridus* So3.2 isolated from the Antarctic rhizosphere soil at the South Shetland Islands, as a novel bioemulsificant/biosurfactant-producer. We report that this biotechnological product from *S*. *luridus* So3.2 has a great capacity to emulsify and displace different oils/hydrocarbons and could be further applied in several industries including cosmetics, textiles, varnishes, pharmaceuticals, mining, and oil.

## Materials and methods

### Collection of soil samples

**Fig 1:**
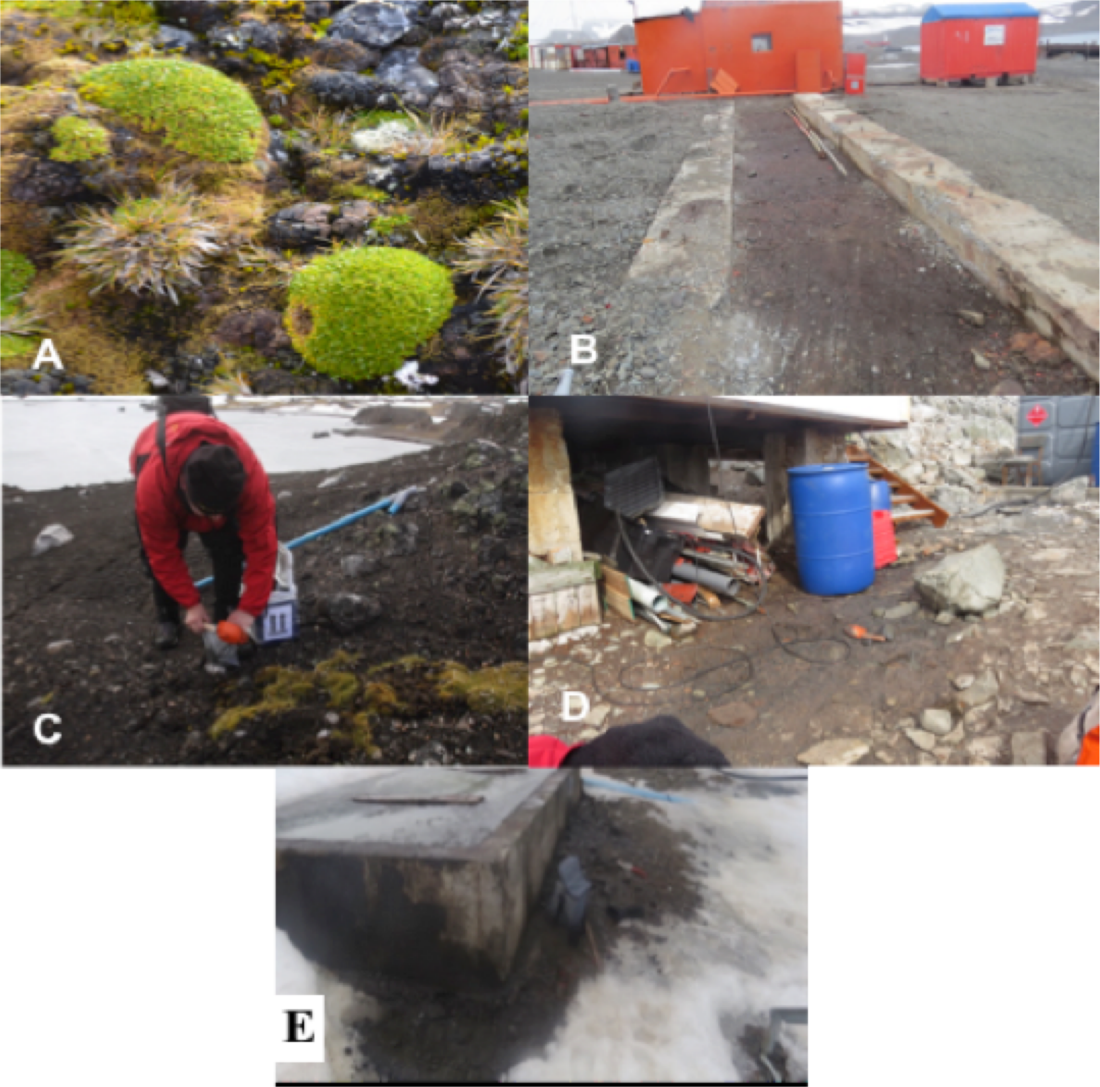
Different sampling sites. A: Peninsula Byers (*Colobanthus quitensis* rhizosphere); B: Fildes Bay-Julio Escudero Base; C: Robert Island (*Deschampsia antarctica* rhizosphere); D: Doumer Island-Yelcho Base; E: Fildes Bay-Escudero Base.

Sampling was carried out in February 2016, and 16 soil samples were collected from South Shetland Islands. Corresponding permission for access, sampling and investigation were conceded by the Instituto Antártico Chileno (Antarctic Chilean Institut-INACH). In this study, five sites were selected, as depicted in Fig 1. The selected sites for sampling were: a) sectors with a permanent human presence, corresponding to the scientific and military bases (Doumer Island-Yelcho Base and Fildes Bay-Escudero Base), which were called impacted places, and b) sectors located within Antarctic Specially Protected Areas (ASPA), which were called pristine places (Peninsula Byers and Robert Island). All soils samples (100 g) were collected at a depth of 10 cm, and kept cold at 4°C for further analysis.

### Isolation of bacteria

For bacterial isolation, 0.1 g of soil was aseptically weighed and homogenized in 900 μl of sterile physiological water (SPW) (0.9% NaCl) for 15 min in a horizontal agitator. A tenfold dilution series was then performed in media: M1 (peptone 2.0 gl^-1^; yeast extract 4.0 gl^-1^; starch 10.0 gl^-1^; agar 18.0 gl^-1^ and pH 7.0 [38], and incubated at 4 and 15°C for 7 days. Each bacterial colony displaying different characteristics was isolated. The purified cultures were maintained at -80°C in glycerol (30%).

### Screening of surface active biomolecules production

Hemolysis on blood agar was tested for 59 bacterial isolates to determine their ability to produce surface-active biomolecules. Hemolysis-positive strains were then evaluated by the oil displacement test and droplet collapse method.

#### Hemolytic activity

The hemolytic activity assay was performed in 5% sheep blood agar plates in triplicate, incubated for 72 h at 15°C. The plates were visually inspected for clear areas (hemolysis) around the bacterial colonies. The diameter of the clear zone is a qualitative method used as an indicator of biosurfactant and bioemulsifier production [11].

#### Drop-collapse test

Promising strains were cultivated in Bushnell Haas media supplemented with different carbon sources (n-hexadecane, olive oil, glucose or glycerol), incubated in shaken flasks at 180 rpm, 30°C, during seven days. Each cell-free supernatant was then obtained and pipetted onto the surface of Parafilm paper. The flattening of the droplet and the diffusion of the drop were followed for seconds or minutes, until changes were observed. Subsequently, 1 μL of methylene blue (which has no influence on the shape of the droplets) was added. The droplet was allowed to dry and the diameter of the drying droplet was recorded [39].

### Morphological and molecular characterization of the bacterial strain

#### Bacterial identification

The bacterial strain was characterized by a combination of phenotypic tests as indicated by Lo Giudice [3], which are based mainly on the determination of colony morphology, reaction to Gram staining, and colony pigmentation. In addition, biochemical tests were performed using the APIZYM kit (Biomerieux, France) according to the manufacturer's instructions.

#### Molecular identification

Genomic DNA was extracted using the UltraClean® Microbial DNA Isolation Kit (MOBIO, CA, USA) according to the manufacturer’s instructions. 16S rDNA was selectively amplified from genomic DNA by polymerase chain reaction (PCR) using universal primers 27f (5’-AGAGTTTGATCCTGGCTCAG-3’) and 1492r (5’-GGTTACCTTGTTACGACTT-3’), enabling the amplification of approximately 1.500bp of the 16S rRNA gene. PCR amplification was performed in a Multigene Optimal Thermal Cycler (Labnet, USA) in 50 μl of PCR mix comprising 25 μl mix reaction buffer 2x (SapphireAmp Fast PCR Master Mix, Takara), 22 μl ultra-pure water, 1 μl of each primer (10 μM) and 1 μl of DNA. The temperature and cycling conditions were as follows: preheating at 94°C for 2 min; 30 cycles at 94°C for 1 min; 55°C for 1 min; 72°C for 1.5 min; and incubation at 72°C for 10 min. The presence of PCR products were assessed by electrophoresis on a 1% agarose gel stained with gel red. Sequencing was done with a dye Terminator Cycle Sequencing Kit and an ABI 3730XL DNA Sequencer (Applied Biosystems) by Macrogen (Korea). The nearest taxonomic group was identified by 16S rDNA nucleotide sequence BLASTN (http://www.ncbi.nlm.nih.gov/blast) using DDBJ/EMBL/GenBank nucleotide sequence databases.

A phylogenetic tree was constructed in MEGA7 [40] using the neighbor-joining method [41]. The bootstrap consensus inferred from 1000 replicates [42] was taken to represent the evolutionary history of the taxa analyzed [42]. The evolutionary distances were computed using the maximum composite likelihood method [43]. The nucleotide sequence identified in this study was deposited in the NCBI nucleotide sequence database (GenBank/NCBI) under accession number MH070262.

### Evaluation of the production of surface active molecules

#### Preliminary evaluation of the surface-active biomolecules production

After screening tests, the most promising bacterial strain (*Streptomyces* So3.2) was evaluated for the production of surface-active biomolecules using two different medias Bushnell Haas (BH) (Becton, Dickinson & Company, Sparks, USA) and Luria Bertani Broth (LB), in order to select the best culture condition for the production of the surface-active biomolecule. For that purpose, 250 ml flasks containing 100 ml of respective culture media were enriched with the carbon sources (2% w/v of olive oil, glycerol, glucose, n-Hexadecane) and inoculated with the bacterial strain, followed by incubation at 180 rpm and 30°C during seven days. The superficial activity of cell free supernatants was then carried out using the drop collapse, oil displacement and emulsification with olive oil as a hydrophobic compound.

#### *Streptomyces* growth kinetics assessment

The bacterial growth kinetics was determined in the culture conditions that allowed greater biosurfactant/bioemulsificant activities on previous assays (BH and LB, both supplemented 2% n-Hexadecane and incubated at 30°C), since it is assumed that those conditions will permit an increased production of bioactive molecules. Growth was quantified by absorbance measurement at 600 nm for 120 h, and converted to cell dry weight (g/l) through a calibration curve. Specific growth rate for the exponential phase (μ) was calculated by the following Eq (1).

$$\mu =\frac{\mathrm{dx}}{\mathrm{dt}}\times \frac{1}{x}$$ (1)

μ **=** specific growth rate (h^-1^)
x = biomass concentration (g/L)
t = time (h)

### Evaluation of the surface-active biomolecules activity

#### Oils and hydrocarbons used for emulsification index (E24)

From each cell-free supernatant, 4 mL were collected and mixed with 4 mL of different oils (olive, soybean, coconut, palm, macauba, mineral) and petroleum of different grades according to the American Petroleum Institute (APIa: high 33.9°; APIm: medium 29.2° and APIb: low 17.3°) in a 10 ml glass tube, mixing well with the vortex for 4 min. The mixture was allowed to stand for the next 24 hours at room temperature, except for the coconut, palm, and macauba oil that were kept at 30°C. The emulsifying activity was expressed as the percentage of the emulsion layer height (cm) divided by the total of the liquid column height after 24 hours [44]. Supernatant from each bacterial strain was evaluated in triplicate. Eq (2) was used to determine the emulsification index (E24).

$$E24=\frac{\mathrm{Heigh}\;\mathrm{to}\;\mathrm{emulsion}\;\mathrm{formed}}{\mathrm{Total}\;\mathrm{height}\;\mathrm{of}\;\mathrm{solution}}\;x\;100$$ (2)

#### Oils and hydrocarbons used for displacement test

Distilled water (40 mL) was deposited on a Petri dish (15 cm of diameter) and then 15 μl of different oils (olive, soybean, coconut, palm, macauba, mineral) and petroleum of different grades (APIa: high 33.9°; APIm: medium 29.2° and APIb: low 17.3°) were deposited on the surface of the water. Finally, 10 μl of each cell free supernatant was added to the oil surface. Tween 80 was used as a positive control. The results were expressed as diameter (cm) of halos displacement obtained after 3 seconds. Each test was done in triplicate [45].

#### Surface tension determination

Cell-free selected supernatants (100 μl) were analyzed on a goniometer Krüss DSA100 (Model: DE 3210) to determine the surface tension (ST) by the drop shape method, as described by Song and Springer (1996) [45].

### Bioemulsifier chemical composition analysis

#### TLC analysis

Thin layer chromatography was performed for crude extract purification in order to obtain the surface-active biomolecules. Plates were prepared using silica gel as the stationary phase. The dried sample was loaded and run with chloroform: methanol (9:1 v/v) as mobile phase. TLC plates were then exposed to an UV light to visualize the spots, which were then purified and evaluated for the displacement test. Another TLC was performed for the chemical determination of carbohydrates (chloroform acetic acid: water 60:30:10) and lipids (chloroform: methanol: water 65:25:4). The resultant spots on the TLC were visualized by spraying H_2_SO_4_ (50%) for carbohydrates, iodine for lipids, and ninhydrin for peptides.

#### Determination of macromolecules by infrared spectroscopy (IR-FT)

The determination of macromolecules conformation was carried out using the infrared spectrum. An aliquot of the most active molecule was deposited in the equipment (Agilent Technologies Cary 630 IR-FT) measuring the transmittance between the frequencies 4000 to 500 cm^-1^.

#### LC–MS analysis

The purified sample was analyzed on a Micromass Q-TOF spectrometer (Applied Biosystems/MDS Sciex 3200). The result was determined by Electroscopy Ionization (ESI) in positive mode.

## Results and discussion

### Collection of soil samples

Fifty-nine strains were isolated from the five sampled sites in this work (Table 2). Our results are similar to others previously reported, where authors have described a greater bacterial biodiversity on the impacted areas due to the human activities [46–48].

**Table 2:** Sampling sites and number of bacterial strains isolated.

N°	Code	Place	Coordinates		Characteristics	N° of isolated bacteria
**1**	So1	Byers Peninsula	62°39'37"S	60°59'6.3"W	ASPA 126^a^, pristine (rhizosphere of *Colobanthus quitensis*)	14
**2**	So2	Fildes Bay	62°12'03.4”S	58°57'40.4”W	Impacted (Chilean Base Julio Escudero)	8
**3**	So3	Robert Island	62°22'23.3"S	59°42'53.2"W	ASPA 112^b^ pristine (rhizosphere of *Deschampsia antarctica*)	9
**4**	So4	Doumer Island	64°54'08.7”S	63°38'36.4”W	Impacted (Yelcho Base)	8
**5**	So9	Fildes BayEscudero Base	62°12'10.8"S	58°57'56.1"W	Impacted (hydrocarbon collector)	20

So: Soil sample; S: South; W: West.

^a^ASPA 126: Byers Peninsula, Livingston Island, South Shetland Islands.

^b^ASPA 112: Coppermine Peninsula, Robert Island, South Shetland Islands.

### Screening of surface active biomolecules production

Hemolytic activity on blood agar reveals halos of hemolysis caused by the rupture of the erythrocyte membrane medium due to the secretion of compounds with surfactant properties [49,50]. Three strains among 59 isolated microorganisms produced hemolytic activity (S1 Table, in Supporting Information). The qualitative test of drop collapse and oil displacement realized with the cell-free supernatant of these three bacterial strains are shown in Table 3. It was observed that the bacterial strain So3.2, isolated from Robert Island rhizosphere soil, generated the greatest activity among the three tested strains.

**Table 3:** Selection of bacterial strains producing active surface molecules.

N°	Code	Oil displacement (olive oil) cm	Drop collapse
**1**	So2.5	0±0	-
**2**	So3.2	10±0.5*	+
**3**	So4.7	8±0.1*	-

*SD n = 3

(+ Positive response - negative response).

### Morphological, phenotypic, and molecular characterization of the bacterial strain

Through morphological and phenotypic characterization, we determined that strain So3.2 is rod-shape Gram-positive bacteria with positive hemolytic activity; colonies on M1 agar were white, round, small, and rough, with white-colored aerial mycelium and no Melanin pigmentation. These results are similar to those described by other authors for bacterial strains belonging to the genus *Streptomyces* [51–53].

The enzymatic characterization by the ApiZym test (as shown in Table 4) shows the production of different enzymes for each immobilized substrate evaluated by the aforementioned kit. It should be noted that some authors highlight the enzymes produced by *Streptomyces* species as being of high biotechnological, clinical, and industrial interest [54–56]

**Table 4:** Reactions of type strain in the APIZYM test.

Enzyme	Reaction	Enzyme	Reaction
**Phosphatase alkaline**	+	Naphthol-AS-BI-phosphohydrolase	+
**Esterase (C4)**	+	α-Galactosidase	+
**Esterase lipase (C8)**	+	β- Galactosidase	-
**Lipase (C14)**	-	β-Glucuronidase	-
**Leucine aminopeptidase**	+	α- Glucosidase	-
**Valine aminopeptidase**	-	β- Glucosidase	+
**Cystine aminopeptidase**	-	N-acetyl-β- Glucosaminidase	-
**Trypsin**	-	α- Mannosidase	-
**α- Chymotrypsin**	-	α- Fucosidase	+
**Phosphatase acid**	+	Control	-

+: positive, -: negative

**Fig 2:**
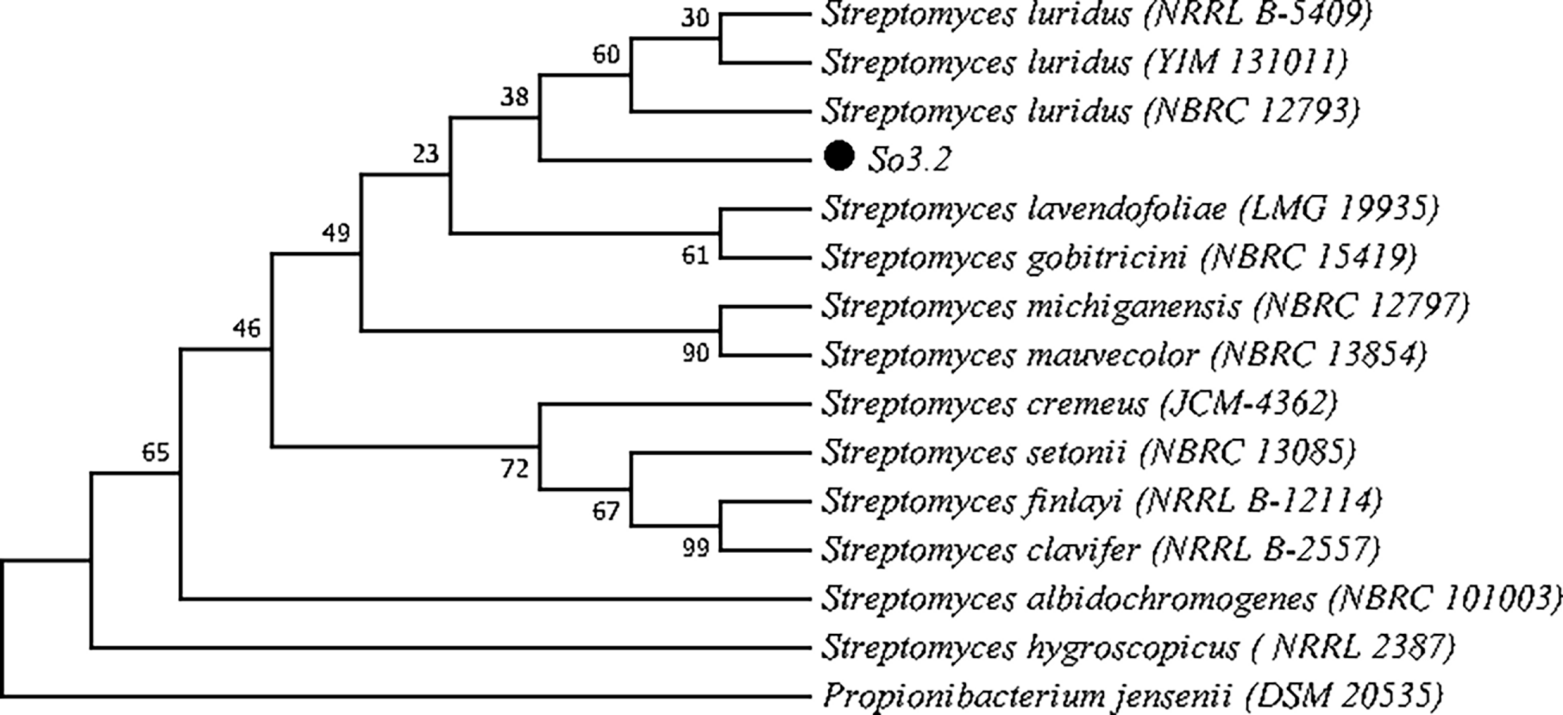
Neighbor-joining dendrogram based on 16S rRNA sequence of strain So3.2 and type strains from *Streptomyces* genus. The optimal tree with the sum of branch length = 0.17933430 is shown. The percentage of replicate trees in which the associated taxa clustered together in the bootstrap test (1000 replicates) are shown next to the branches. The evolutionary distances were computed using the Maximum Composite Likelihood method and are in the units of the number of base substitutions per site.

The molecular identification of strain So3.2 through the sequencing of the 16s rRNA gene resulted in a clear phylogenetic relation with the Actinobacteria phylum and the closest identity relation with the species *Streptomyces luridus*, showing 99% of identity according to the Basic Local Alignment Search Tool (BLAST) and clustering together with *S*. *luridus* type strains as shown in Fig 2.

#### Evaluation of the surface-active biomolecules production from *Streptomyces luridus*

Evaluation of the production of bioactive compounds from *S*. *luridus* strain was carried out in BH media supplemented with different carbon sources and LB without supplementation, while activity of the bioactive compounds was verified by analyzing the drop collapse, oil displacement, and emulsification with olive oil as a hydrophobic compound, and the measurement of surface tension in cell-free supernatants (Table 5).

**Table 5:** Evaluation of the surface-active biomolecules production by *Streptomyces luridus* So3.2 on different culture media and carbon sources.

Culture medium	Oil displacement (olive oil) cm	Drop collapse H_2_O	Emulsification (olive oil)	Surface tension (mN/m)
Bushnell Haas n-Hexadecane 2%	15±0.5*	+	+	56.85 ± 0.58
Bushnell Haas olive oil 2%	10±0.2*	+	+	67.31 ± 1.49
Bushnell Haas glucose 2%	0±0	-	-	71.20 ± 1.59
Bushnell Haas glycerol 2%	0±0	-	-	66.95 ± 0.21
LB	14±0.5*	+	+	58.43 ± 0.43
H_2_O	0±0*	-	-	72.88 ± 0.48

*SD n = 3

(+ Positive response – Negative response).

Supernatants of LB and Bushnell Haas (BH) medium grown with 2% n-Hexadecane were the most active in the physicochemical tests and olive oil was also able to stimulate some production of biosurfactant. These results are similar to those reported in [57] who observed that the use of glycerol and glucose (hydrophilic molecules) as carbon sources did not stimulate the production of surface-active molecules such as biosurfactants. Furthermore, Zambry demonstrated that glycerol used as the only source of carbon in culture media is not appropriate for the production of surfactant biomolecules by bacterial strains belonging to the genus *Streptomyces* [58]. Additionally, the same work demonstrated that a strain of *Streptomyces* has the ability to produce biosurfactants through stimulation with olive oil. Therefore, it is possible to conclude that hydrophobic carbon sources are better than hydrophilic ones for the production of surface-active biomolecules by *S*. *luridus* S0 3.2.

#### Growth of *Streptomyces luridus* strain

**Fig 3:**
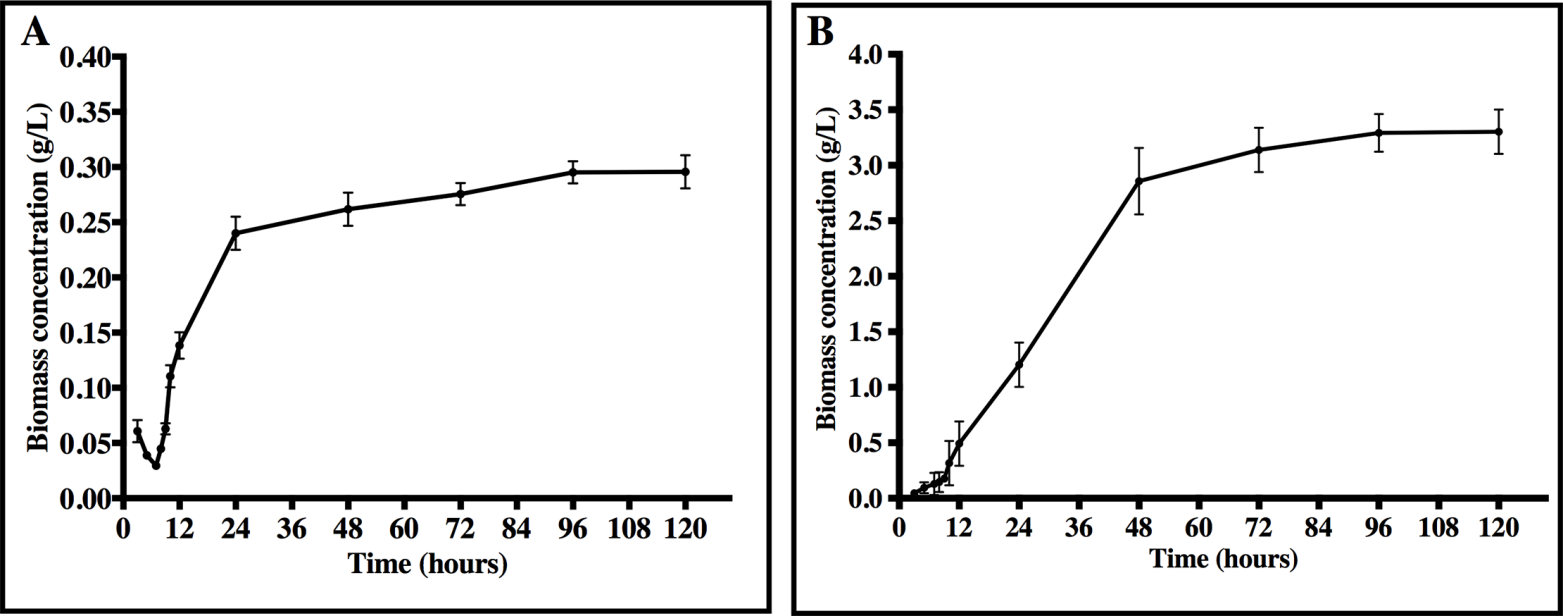
Growth kinetics of the strain So3.2 in BH media with n-Hexadecane (A) or LB media (B). Data are given as the mean ± SD for n = 3 samples.

The growth kinetic of *Streptomyces luridus* So3.2 described in Fig 3, showed that the stationary phase of microbial growth was reached in around 30 hours for the BH medium supplemented with n-Hexadecane. As for the LB medium, the stationary growth phase was reached in around 40 hours. The calculated specific growth rate (μ) was 0.32 h^−1^ and 0.24 h^−1^ for BH medium with n-Hexadecane and LB medium, respectively. These results are similar to those reported by Cox, 2004 for *Streptomyces coelicolor*, where the growth rates were determined at 30°C, ranging from 0.02 h ^-1^ to 0.30 h ^-1^ [59].

### Evaluation of the surface-active biomolecules activity

**Fig 4:**
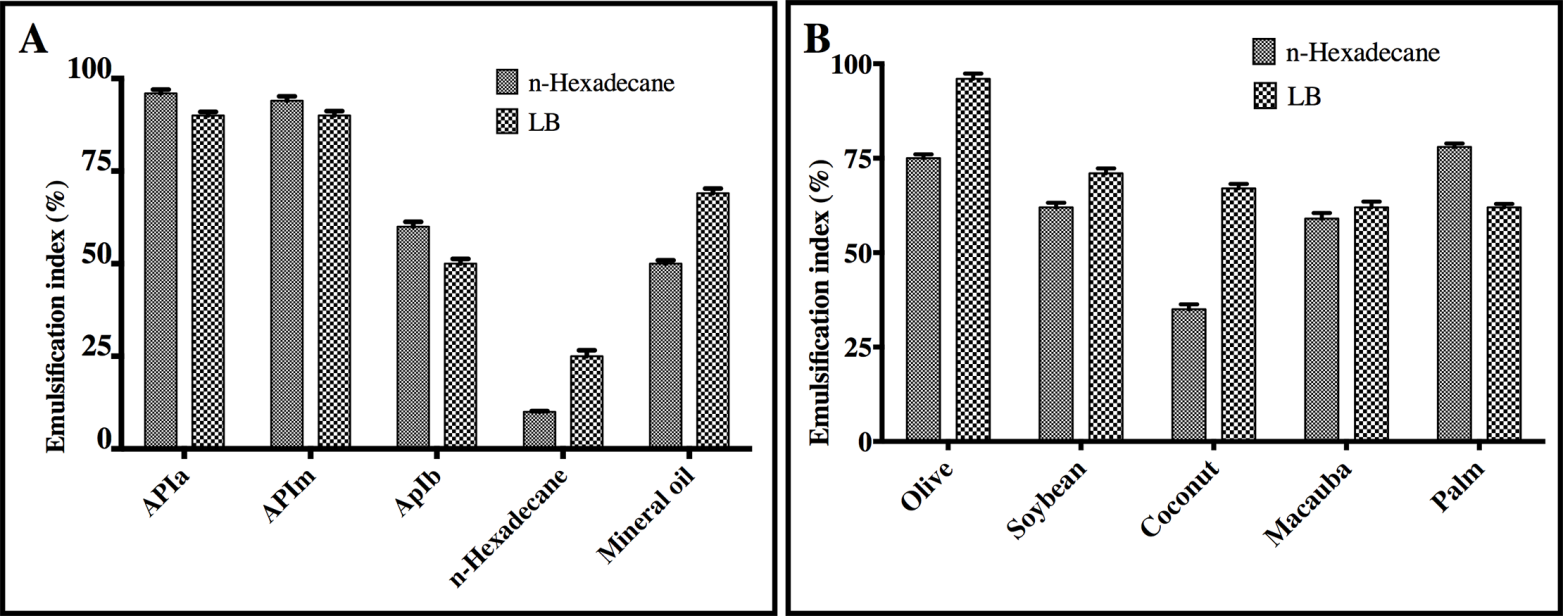
Emulsification index (E24) produced by supernatant of the strain *Streptomyces* So3.2 in BH with n-Hexadecane and LB culture medium. In A, against different hydrocarbons. In B different oil results are shown. Data are given as the mean ± SD for n = 3 samples.

Cell free supernatants obtained from cultures on LB and BH media with n-Hexadecane at 96 hours of fermentation were subjected to displacement and emulsification index, assessment using different petroleum hydrocarbons, n-Hexadecane and mineral oil (Fig 4A) and vegetable oils (Fig 4B). Supernatants obtained with n-Hexadecane (in grey) showed an emulsification index higher than 90% in high and medium grade oil. On the other hand, for low grade petroleum and mineral oil, the emulsification index did not exceed 60%. In addition, we observed that the supernatant showed little effect when emulsifying n-Hexadecane. Likewise, LB supernatant (black and white with patterns) showed similar results, except for the superior activity in mineral oil and n-Hexadecane. Regarding emulsification of vegetable oils, we observed that LB supernatants have higher activity when compared to HB media with n-Hexadecane supernatants, reaching over 60% in all oils tested and up to 98% in olive oil (Fig 4B).

Compared to other studies for *Streptomyces* strains, our emulsification results showed that the supernatants obtained with various carbon sources have good emulsifier production capacity, being able to emulsify different amphipathic compound [60,61]. Additionally, the emulsification index results for hydrocarbons assessed in this work were similar to those published by [57]. An emulsification index of 80% was reported for petroleum, according to Zambry et al. [58], similar to those obtained in our work for oils of grades APIa 33.9° and APIm 28.6°, where an emulsification index of 90% was reached. With these results, we highlight the carbon source n-Hexadecane as a good substrate for the production of surface active biomolecules using the Antarctic strain *Streptomyces luridus*, with a high emulsification capacity for both oils and vegetable oils. In addition, the surface-active biomolecules in its supernatant generate emulsifications stable in 24 hours at room temperature with an activity of over 50% in the evaluated oils. This particular characteristic is important since it benefits the lifetime of the product as well as the storage of the bioemulsifier (Patil & Chopade) [62].

### Oils and hydrocarbons used for displacement test

**Fig 5:**
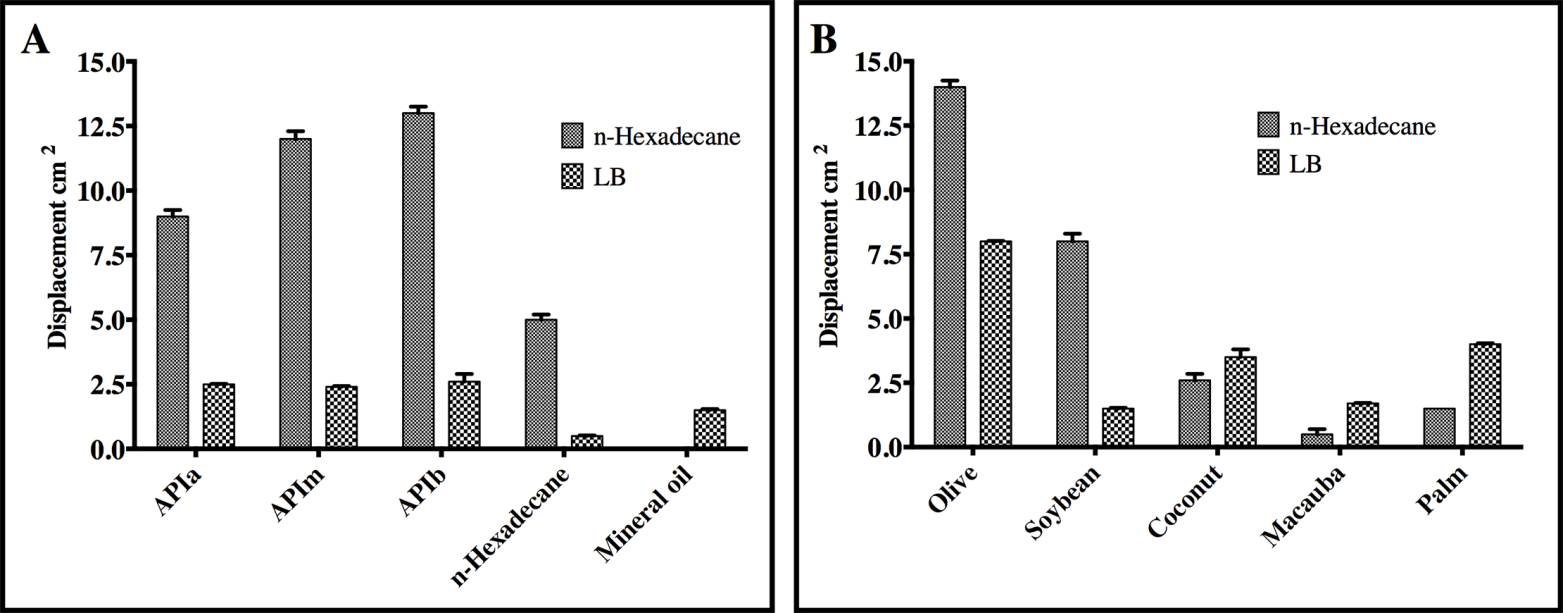
Displacement activity of the supernatants produced by the strain *Streptomyces* So3.2 in the BH with n-Hexadecane and LB culture medium. In **A** different hydrocarbons and in **B** different oils. Data are given as the mean ± SD for n = 3 samples.

The displacement results, obtained by the application of the supernatants on different hydrocarbons and vegetable oils, are shown in Fig 5. The maximum activity observed in hydrocarbons (Fig 5A) was obtained with supernatants of BH (with n-Hexadecane) in the petroleum (APIb) with a 12 cm halo. We can also observe that the activity disappears in the mineral oil, which is a liquid byproduct of the distillation of the oil from the crude oil. This low displacement activity could be justified by the mineral oil composition, typically of alkanes (typically 15 to 40 carbons) and cyclic paraffin. The observed displacement in the application of the supernatant obtained with LB medium was lower, being around 3 cm for the three different types of oil (APIa, APIb and APIm). In addition, LB supernatants present scant activity with mineral oil, and almost no activity with n-Hexadecane.

In the case of oily halos (displacements) formed by the application of the supernatants (Fig 5B), the maximum activity was measured for olive oil with 14 cm displacement produced by supernatant BH (n-Hexadecane). We can also observe that the activity decreases in the oils of soybean, coconut and palm, especially in oil of Macauba, not exceeding 2 cm of halos formed. Possibly, the composition of these oils (from 16C to 18C) influences the displacement activity. Regarding LB supernatants (Fig 5B, in black and white with patterns), the maximum activity was 8 cm for olive oil and 5 cm for macauba oil. Comparing these results, it is possible to conclude that the tested supernatants, obtained from BH (with n-Hexadecane) and LB culture medias, have very different displacements activities.

Our results for displacement test with *S*. *luridus* So 3.2 supernatants are similar and in some cases greater than others previously reported. For example, for *Nocardiopsis alba* the displacements of olive oil reached a maximum of 6 cm [63]. In addition, the strain *Streptomyces gedanensis* (LK-3), which was evaluated in crude petroleum, produced average maximum displacement halos of 2 cm [64]. Likewise, a study evaluated five different *Streptomyces* isolated from soil, where the supernatants obtained showed that the displacement activity in crude oil did not exceed 1.5 cm [37].

For LB supernatant displacement results in the three oils, we can observe that the displacement activity does not exceed 3.5 cm. These results are the inverse to those obtained in the emulsification indexes with the same supernatants; this phenomenon may be due to the different sources of carbon and nitrogen present in each media. This could change the amount and type of bioemulsifier produced by our strain. This characteristic of altered grades of surface activity has been described by several authors for different bacteria such as *Pseudomonas* [65], *Bacillus* [66] and Actinobacteria [67]. With this work, we highlight the activity of the supernatants generated by BH media with n-Hexadecane supplementation for the strain *Streptomyces luridus* So 3.2 (S1 Fig), where a displacement halo of 8 cm in diameter was formed in APIa grade petroleum, which is larger than those reported in similar works [37,64].

The hemolytic assay described in this work may be a good choice as a tool for assessing bacterial strains capable of producing surface active biomolecules [68]. On the other hand, Tugrul et al. [69], reported that the drop collapse method may be used to detect biosurfactant-producing microorganisms in natural environments. In addition, emulsification and oil displacement methods are interesting tools to assess the production of surface-active biomolecules in the supernatants. The results obtained in this work with the bacteria Antarctica *S*. *luridus* showed that the supernatants have positive activity confirmed by drop collapse, oil displacement, hemolysis tests, and use of the emulsification index, as described by Cooper and Goldenberg [70].

In addition, good emulsification and oil displacement activity of hydrocarbons and oils were shown, without the reduction of surface tension, as Uzoigwe et al. also reported [19]. The ability to reduce surface and interfacial tension stands for 20 the distinctive contrast between biosurfactant and bioemulsifiers. These molecules can both form stable emulsions but it is still unclear why bioemulsifiers do not show significant changes in surface/interfacial tension between different phases (liquid-air, liquid-liquid, liquid-solid). Some studies indicate that emulsification index and emulsification activity are screening tests for measuring the emulsification capacity of any surface active molecule with different hydrocarbons, as described by Jagtap et al. [71]. Likewise, using the index emulsification, Viramontes-Ramos et al. [72] identified six isolates that could efficiently emulsify different hydrocarbons (more than 50% against diesel, decane, kerosene and motor oil) without showing a significant reduction in surface tension of their culture broths. In addition, the emulsifying potential of the surface-active compounds from different yeasts showed that no reduction in surface tension has been evaluated using the emulsification index test by Monteiro et al. [73].

With this background, we infer that the strain *Streptomyces luridus* So 3.2 is a bioemulsifer producer. From the point of view of applications, surfactant biomolecules such as biosurfactants, play a role in reducing surface tension, while the bioemulsifiers are involved in the formation and stabilization of emulsions and have surfactant and emulsifying properties that contribute to their broad functions that are unique in industrial and biotechnological uses, among others [74]. The molecules produced by bacterial strain 20 isolated in this work could succeed in different applications, especially in the hydrocarbon cleaning processes such as petroleum. In addition, since it is an unreported Antarctic *Streptomyces* strain producing bioemulsifiers, a completely new field for the characterization and purification of these possibly new biomolecules is suggested.

### Bioemulsifier chemical composition analysis

#### TLC analysis

**Fig 6:**
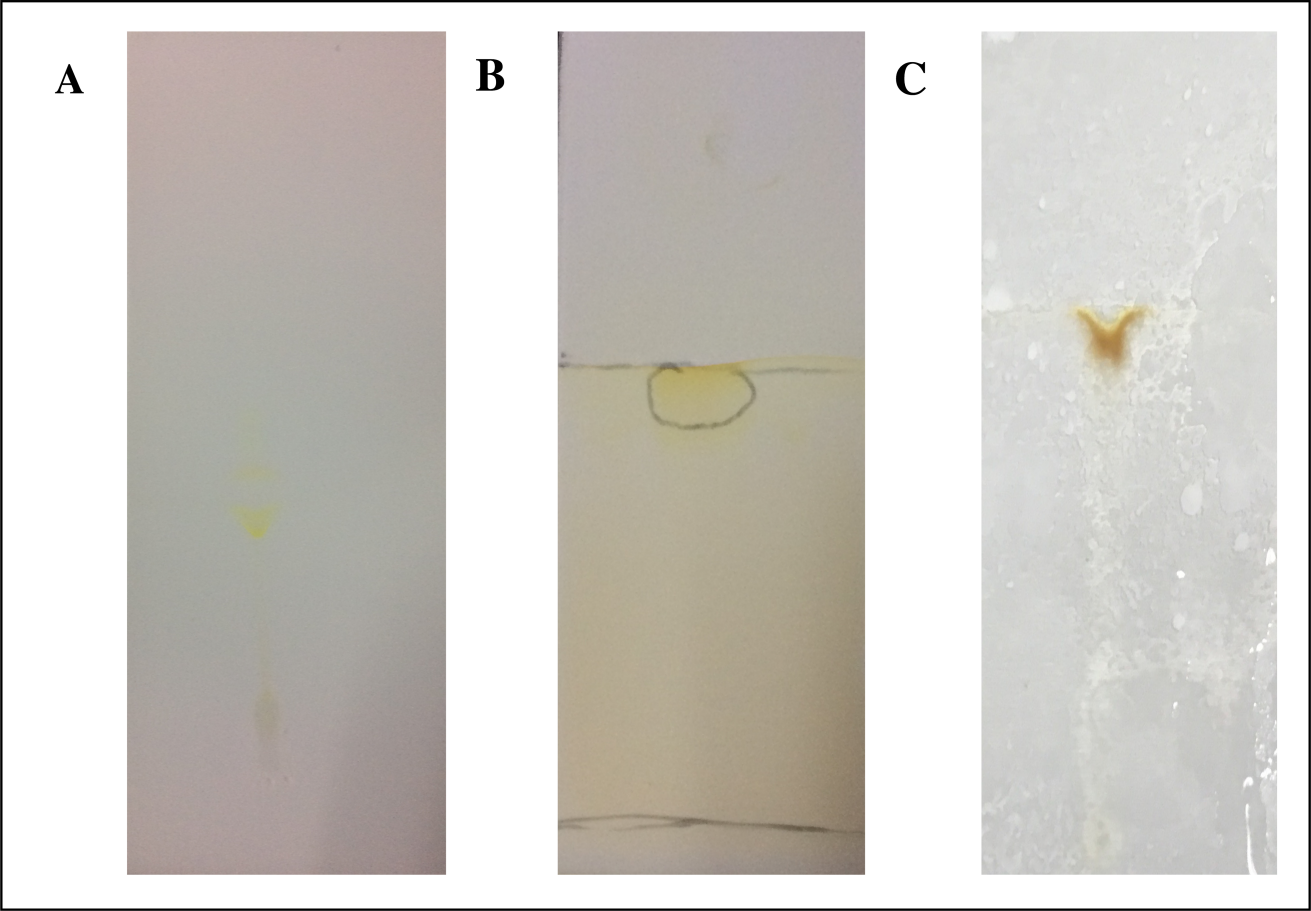
TLC assays of chemical composition of the purified bioemulsifier of *S*. *luridus*. (A) Ninhydrin, (B) Iodine, (C) H_2_SO_4_.

The results of the purified bioemulsifier chemical composition analysis are shown in Fig 6. It can be observed that the compound does not show presence of peptides. However, carbohydrates and lipids on its compositions were confirmed by reaction to H_2_SO_4_ and iodine, respectively.

#### Determination of macromolecules by infrared spectroscopy (IR-FT)

**Fig 7:**
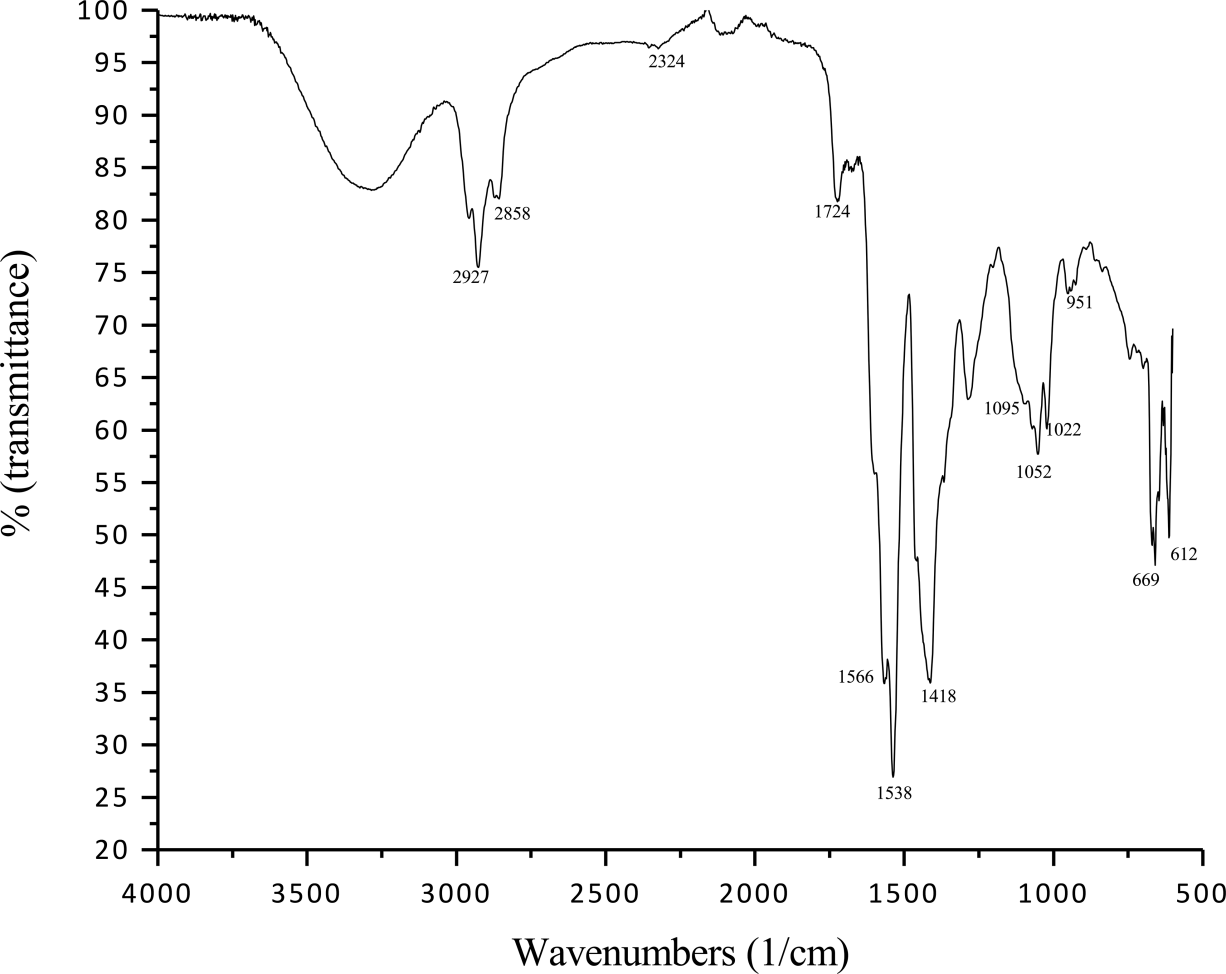
IR-TF spectrum of the purified bioemulsifier of the strain *Streptomyces luridus* So3.2.

Analysis of the purified compound by infrared spectroscopy is shown in Fig 7. The most important peak was located at 2927 cm^−1^, corresponding to the bioemulsifer produced by *S*. *luridus*. Bending vibrations of methyl group are shown at 1724 cm^−1^; stretching of the C = O double bond of esters and carboxyl groups at 1566 cm^−1^ and C-H (fatty acids) peaks were revealed between 900 and 500 cm^−1^, which evidence CH_2_CH_3_ stretch and CO stretch, probably corresponding to a fatty acid.

#### LC–MS analysis

**Fig 8:**
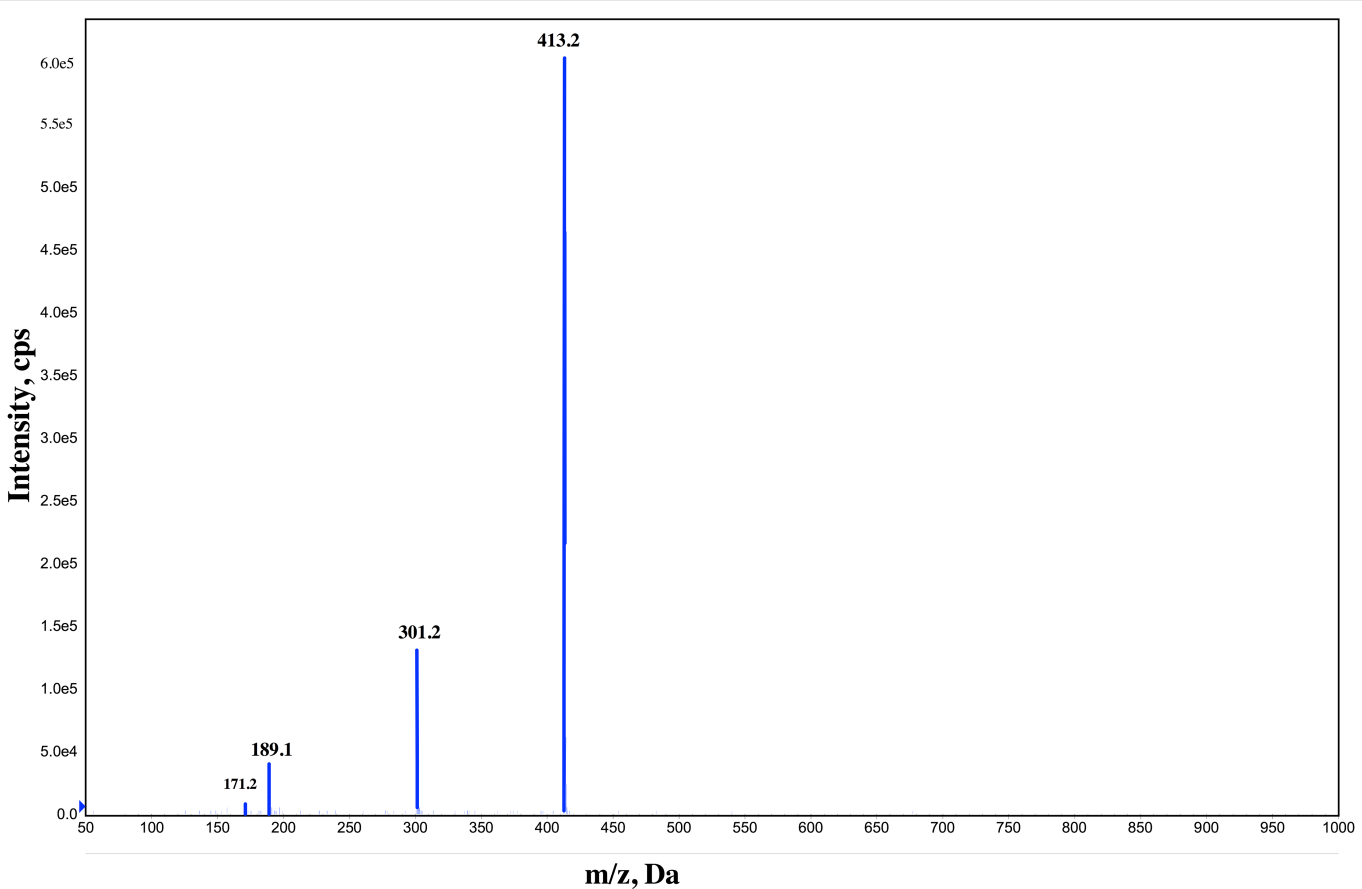
Mass spectroscopy (LC-MS) spectra of the purified bioemulsifier from *Streptomyces luridus* So3.2 strain.

The result of the LC-MS (Fig 8) revealed an approximation of the molecular weight of the biosurfactants produced by *Streptomyces luridus* So3.2. According to electro-nebulization in positive mode, three main peaks were detected at 413.5, 301.5 and 189.1, which allows us to conclude that the molecular weight of the bioemulsifier would be 413.5.m/z. Additionally, it was determined that no trace of protein was present in the product.

## Conclusions

This study showed that it is possible to isolate bacteria from impacted and pristine soils from different locations in Antarctica. Screening for the production of active surface biomolecules, by cultivating blood agar, showed that strain So3.2 isolated from a sample of rhizosphere soil on Robert Island has a high hemolytic activity. The identification of the bacterial strain by molecular markers resulted on *Streptomyces luridus* species as the nearest taxa. This microorganism showed the ability to produce active surface biomolecules in BH and LB culture media with n-Hexadecane as a carbon source. The produced molecules could emulsify and displace different oils and hydrocarbons at high levels. Despite this, the supernatants produced do not have the capacity to decrease the surface tension, as a biosurfactant. Consequently, the studied compound corresponds to a bioemulsifier, which was confirmed by multiple results of oil and petroleum emulsification. In addition, the high molecular mass observed in the LC-MS analysis supports the conclusion that the molecule produced is a bioemulsifier. The chemical composition of the isolated bioemulsifier analyzed by infrared spectroscopy presented characteristics of fatty acid.

The supernatant produced by Antarctic *S*. *luridus* So3.2 could be used as an alternative to chemical surfactants for the bioremediation of oil leakage in aquatic environments. The industries involved in the recovery of nutrients, cosmetics, textiles, varnishes, pharmaceuticals and mining and oil, given its high affinity for emulsion and the displacement of amphipathic molecules. Further investigation is necessary in order to obtain an accurate elucidation of the molecule structure, which subsequently could allow a quantitative determination of its productivity and the optimization the strain culture conditions.

## Supporting information

S1 Fig Displacement test of petroleum APIa grade in water before (A), and after the application of supernatant obtained from BH media with n-Hexadecane (B).
(TIFF)

S1 Table Hemolytic activity of isolated strains. (DOCX)
