# Supplementary figures and images for: Streptomyces luridus So3.2 from Antarctic soil as a novel producer of compounds with bioemulsification potential
Source: PLoS One. 2018 Apr 23;13(4):e0196054. doi: 10.1371/journal.pone.0196054 (PMC5912782; doi:10.1371/journal.pone.0196054)

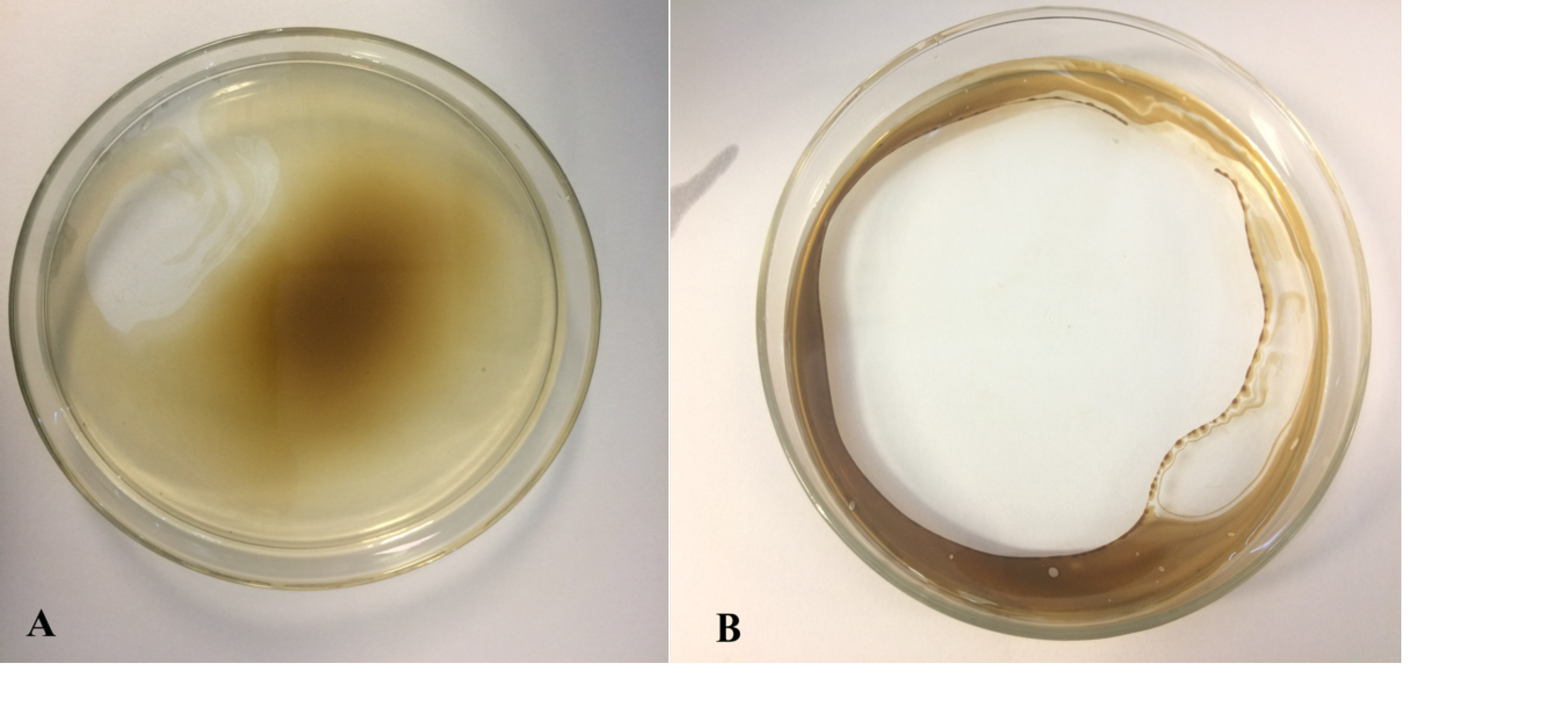

Supplement: S1 Fig — Displacement test of petroleum APIa grade in water before (A), and after the application of supernatant obtained from BH media with n-Hexadecane (B). (TIFF) [file pone.0196054.s001.tiff]
