# Supplementary material for: Streptomyces luridus So3.2 from Antarctic soil as a novel producer of compounds with bioemulsification potential
Source: PLoS One. 2018 Apr 23;13(4):e0196054. doi: 10.1371/journal.pone.0196054 (PMC5912782; doi:10.1371/journal.pone.0196054)
Supplement: S1 Table — (DOCX) [file pone.0196054.s002.docx]

**Supporting information**

**S1 Table. Hemolytic activity of isolated strains**.

| **Nº** | **Laboratory identification** | **Sampling sites** | **Hemolytic activity** |
| --- | --- | --- | --- |
| 1 | So1.1 | Byers Peninsula | - |
| 2 | So1.2 | Byers Peninsula | - |
| 3 | S01.3 | Byers Peninsula | - |
| 4 | So1.4 | Byers Peninsula | - |
| 5 | So1.5 | Byers Peninsula | - |
| 6 | So1.6 | Byers Peninsula | - |
| 7 | So1.7 | Byers Peninsula | - |
| 8 | So1.8 | Byers Peninsula | - |
| 9 | So1.9 | Byers Peninsula | - |
| 10 | So1.10 | Byers Peninsula | - |
| 11 | So1.11 | Byers Peninsula | - |
| 12 | So1.12 | Byers Peninsula | - |
| 13 | So1.13 | Byers Peninsula | - |
| 14 | So1.14 | Byers Peninsula | - |
| 15 | So2.1 | Fildes Bay | - |
| 16 | So2.2 | Fildes Bay | - |
| 17 | So2.3 | Fildes Bay | - |
| 18 | So2.4 | Fildes Bay | - |
| 19 | So2.5 | Fildes Bay | Yes |
| 20 | So2.6 | Fildes Bay | - |
| 21 | So2.7 | Fildes Bay | - |
| 22 | So2.8 | Fildes Bay | - |
| 23 | So3.0 | Robert Island | - |
| 24 | So3.1 | Robert Island | - |
| 25 | So3.2 | Robert Island | Yes |
| 26 | So3.4 | Robert Island | - |
| 27 | So3.5 | Robert Island | - |
| 28 | So3.6 | Robert Island | - |
| 29 | So3.7 | Robert Island | - |
| 30 | So3.8 | Robert Island | - |
| 31 | So3.9 | Robert Island | - |
| 32 | So4.1 | Doumer Island | - |
| 33 | So4.2 | Doumer Island | - |
| 35 | So4.3 | Doumer Island | - |
| 36 | So4.4 | Doumer Island | - |
| 37 | So4.5 | Doumer Island | - |
| 38 | So4.6 | Doumer Island | - |
| 39 | So4.7 | Doumer Island | Yes |
| 40 | S04.8 | Doumer Island | - |
| 41 | So9.1 | Fildes Bay | - |
| 42 | So9.2 | Fildes Bay | - |
| 43 | So9.3 | Fildes Bay | - |
| 44 | So9.4 | Fildes Bay | - |
| 45 | So9.5 | Fildes Bay | - |
| 46 | So9.6 | Fildes Bay | - |
| 47 | So9.7 | Fildes Bay | - |
| 48 | So9.8 | Fildes Bay | - |
| 49 | So10 | Fildes Bay | - |
| 50 | So11 | Fildes Bay | - |
| 51 | So12 | Fildes Bay | - |
| 52 | So13 | Fildes Bay | - |
| 53 | So14 | Fildes Bay | - |
| 54 | So15 | Fildes Bay | - |
| 55 | So16 | Fildes Bay | - |
| 56 | So17 | Fildes Bay | - |
| 57 | So18 | Fildes Bay | - |
| 58 | So19 | Fildes Bay | - |
| 59 | So20 | Fildes Bay | - |

(-): negative for hemolysis

Yes: positive for β hemolysis

**S1 Fig. Displacement test of petroleum APIa grade in water before (A), and after the application of supernatant obtained from BH media with n-Hexadecane (B).**
